# Design and Fabrication of Multi-Frequency and Low-Quality-Factor Capacitive Micromachined Ultrasonic Transducers †

**DOI:** 10.3390/mi16070797

**Published:** 2025-07-08

**Authors:** Amirhossein Moshrefi, Abid Ali, Mathieu Gratuze, Frederic Nabki

**Affiliations:** Department of Electrical Engineering, École de Technologie Supérieure (ETS), Montreal, QC H3C 1K3, Canada; abidaliqau@gmail.com (A.A.); mathieu.gratuze.1@ens.etsmtl.ca (M.G.); frederic.nabki@etsmtl.ca (F.N.)

**Keywords:** ultrasonic sensor, quality factor optimization, array design, CMUT, MEMS

## Abstract

Capacitive micromachined ultrasonic transducers (CMUTs) have been developed for air-coupled applications to address key challenges such as noise, prolonged ringing, and side-lobe interference. This study introduces an optimized CMUT design that leverages the squeeze-film damping effect to achieve a low-quality factor, enhancing resolution and temporal precision for imaging as one of the suggested airborne application. The device was fabricated using the PolyMUMPs process, ensuring high structural accuracy and consistency. Finite element analysis (FEA) simulations validated the optimized parameters, demonstrating improved displacement, reduced side-lobe artifacts, and sharper main lobes for superior imaging performance. Experimental validation, including Laser Doppler Vibrometer (LDV) measurements of membrane displacement and mode shapes, along with ring oscillation tests to assess Q-factor and signal decay, confirmed the device’s reliability and consistency across four CMUT arrays. Additionally, this study explores the implementation of multi-frequency CMUT arrays, enhancing imaging versatility across different air-coupled applications. By integrating multiple frequency bands, the proposed CMUTs enable adaptable imaging focus, improving their suitability for diverse diagnostic scenarios. These advancements highlight the potential of the proposed design to deliver a superior performance for airborne applications, paving the way for its integration into advanced diagnostic systems.

## 1. Introduction

Ultrasonic imaging stands as a pivotal technology in contemporary diagnostics, valued for its non-invasive application, real-time operation, and ability to produce high-resolution images. It serves crucial roles in diverse fields, from cardiovascular evaluations to prenatal monitoring, providing clinicians with detailed visualizations of soft tissue structures. Nevertheless, achieving consistently superior image quality remains a significant hurdle, often compromised by noise, artifacts, and the inherent trade-offs between image resolution and sensor performance [1,2,3]. Noise is one of the most pressing challenges in ultrasonic imaging, as it can obscure critical features and diminish diagnostic reliability. Sources of noise are varied, including electronic interference, the physical properties of materials, and imperfections in the imaging transducer’s design. Beyond noise, image artifacts such as side lobes further degrade image quality by introducing misleading reflections or blurring vital details. This issue becomes even more pronounced in high-frequency imaging, where accurate visualization of minute structures is imperative [4,5,6,7]. The quality (Q) factor of ultrasonic sensors plays an important role in influencing noise levels and image artifacts. There is an inherent trade-off between resolution and penetration in transducers with varying frequencies and Q. Higher frequencies enhance resolution but also increase attenuation, limiting penetration depth. Conversely, a lower Q broadens the bandwidth, improving axial resolution while potentially degrading surface image quality [1,8,9,10]. By carefully calibrating the mechanical and electrical parameters of these transducers, it is possible to significantly reduce noise and artifacts while sharpening the primary imaging lobe. These advancements ultimately result in more precise imaging, paving the way for reliable and accurate air-coupled diagnoses. Comparing CMUTs to conventional piezoelectric transducers reveals distinct benefits in imaging resolution and ring-down times. Their microfabrication compatibility enables easier integration with on-chip electronics. They also provide broader bandwidth, allowing multi-frequency imaging within a single device. Piezoelectric transducers often require specialized assembly and may exhibit narrower operational ranges. Highlighting these contrasts directly underscores CMUTs’ unique advantages for airborne applications [11,12].

Low Q shortens the ring-down, enabling pulse-echo imaging of objects only a few millimeters away (robotics proximity, flow metering, or gesture recognition), where long ringing would mask near-surface echoes. Multi-frequency capability allows dynamic selection between deeper penetration (longer wavelength, lower attenuation) and fine lateral detail (shorter wavelength). In addition, multi-frequency imaging using CMUTs enhances imaging versatility by enabling operation across a broad frequency range, allowing deeper penetration at lower frequencies and higher resolution at higher frequencies. This adaptability improves the accuracy of soft tissue visualization, vascular assessments, and contrast-enhanced ultrasound techniques while reducing the need for multiple transducers, leading to more compact and cost-effective imaging systems. Also, adaptive resolution techniques dynamically adjust imaging parameters based on real-time data, optimizing spatial resolution and penetration depth for different diagnostic scenarios. For instance, lower frequencies can be prioritized for deep tissue imaging, while higher frequencies enhance fine details in high-resolution imaging. These innovations not only enhance imaging quality but also pave the way for intelligent, self-optimizing ultrasound systems capable of delivering personalized and high-precision diagnostics [11,13,14,15,16,17].

Building on these advancements, this paper presents an approach for optimizing CMUT design to further enhance imaging performance in theoretical and fabrication parts, of which the theoretical part is briefly explained in [4]. The primary objective is to develop a CMUT optimized for high-frequency imaging while addressing key performance trade-offs, including achieving a low-quality factor (Q ≈ 2), enhanced displacement, reduced side lobes, and a well-defined main lobe in array configurations. Finite element analysis (FEA) simulations conducted in COMSOL v6.0 confirmed the feasibility of these improvements, demonstrating reduced Q-factor, improved displacement, and effective side-lobe suppression under realistic conditions. The fabricated CMUT arrays were then experimentally evaluated through displacement measurements, mode shape analyses, sensitivity assessments, and ring-down tests, all of which validated the anticipated low-Q-factor performance. Indeed, unlike prior CMUT studies that focus solely on wide bandwidth or multi-frequency operation, our work is the first to leverage squeeze-film damping via an optimized perforation pattern to achieve an ultra-low Q and integrate four distinct center-frequency arrays on a single die for airborne adaptive imaging. This combination yields both fast ring-down (for temporal precision) and tunable spatial resolution across frequencies, which has not been demonstrated in the literature.

The remainder of this paper is organized as follows: Section 2 details the theoretical framework and fabrication process, Section 3 presents simulation and experimental results, and Section 4 concludes with key findings and future perspectives.

## 2. Materials and Methods

### 2.1. Modeling

Optimizing a CMUT requires precise evaluation of its Q-factor, resonance frequency, and membrane displacement. These fundamental parameters are shaped by the device’s damping characteristics and structural properties. The damping coefficient of a perforated CMUT membrane can be described using (1) and (2) [18,19]:

(1) $$\begin{array}{l} C=\frac{3\mu A}{d^{3}}\frac{Nr_{0}^{4}\ln(\frac{4}{\beta })}{\pi k(\beta )}\\ \beta =\frac{r_{0}}{r_{c}};\;k(\beta )=4\beta^{2}-\beta^{4}-4\ln(\beta )-3 \end{array}$$

(2) $$Q=\frac{\omega_{0}m}{C}$$

In these equations, μ refers to the viscosity of the surrounding fluid, while m represents the effective membrane mass, and ω_0_ denotes the resonance frequency. Parameters such as the hole radius (r_0_), cell radius (r_c_), and the total number of perforations (N) influence energy dissipation. Additionally, β is defined as the ratio of the hole radius to the cell radius, with k(β) accounting for fluid dynamics through the perforations, ultimately affecting damping and the Q-factor.

Adjusting the number of holes relative to the diameter allows us to fine-tune the Q-factor. In applications requiring fast temporal resolution (less ringing), a lower Q-factor is desired, and hence a higher perforation density can be beneficial. Conversely, fewer holes might lead to higher output amplitude but at the expense of longer ringing times.

The relationship between membrane displacement and influencing factors is captured in (3) [19]:

(3) $$W_{\max}=\frac{\varepsilon_{0}V^{2}a^{4}}{128Dd^{2}}$$

(4) $$D=\frac{Eh^{3}}{12(1-\nu^{2})}$$

Here, W_max_ represents the maximum displacement, which depends on several variables: the applied voltage (V), the radius of the membrane (a), and the gap (d) between the membrane and substrate. Furthermore, structural properties like flexural rigidity (D), Young’s modulus (E), membrane thickness (h) and Poisson’s ratio (ν) collectively dictate how the membrane responds to applied voltage.

In addition, an effect which has an impact on CMUT performance is the squeeze-film effect. It arises from the thin air gap between the vibrating membrane and the substrate. When the membrane oscillates, the air in the gap is compressed and rarefied, causing energy dissipation due to viscous damping. This dissipation reduces the Q of the CMUT. The energy dissipation can be described using the squeeze-film damping equation, which relates the damping force to the velocity of the membrane. The total energy dissipated per cycle energy dissipation (ED) is proportional to the damping coefficient, inversely impacting the Q, calculated as below.

(5) $$Q=\frac{2\pi .\;\mathrm{Stored}\;\mathrm{energy}}{\mathrm{Total}\;\mathrm{energy}\;\mathrm{dissipated}\;\mathrm{per}\;\mathrm{cycle}}$$

**Figure 1:**
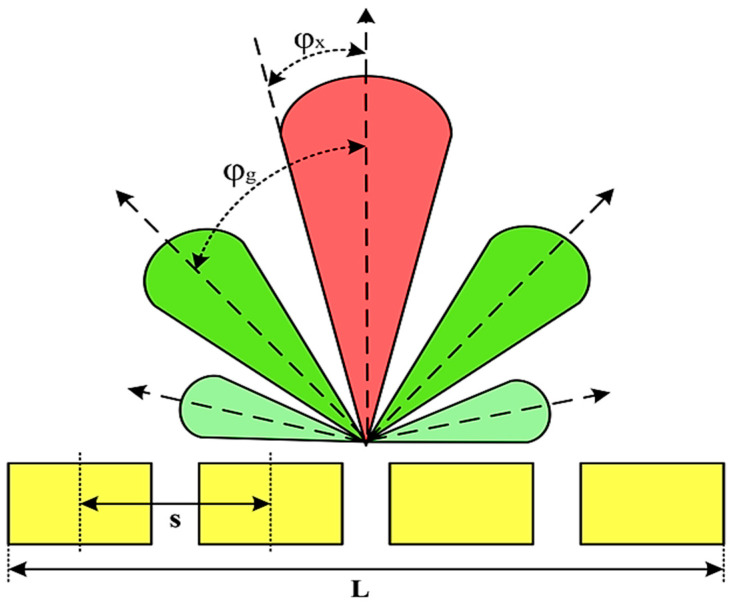
Perspective view of the lobe shape of an array of CMUT. The angular positions of the lobes were calculated based on (6) and (7).

Moreover, array lobes play a crucial role in defining the beam pattern and performance of ultrasonic transducers. Figure 1 illustrates the array lobe patterns generated by the CMUT design. It highlights the main lobe and grating lobes, emphasizing the optimization approach used to suppress side lobes and enhance signal clarity.

As shown in Figure 1, the effect of the array lobes should be investigated. To minimize the grating lobes in an array structure, their angle ($\varphi_{g}$) is given by (6) [20]:

(6) $$\varphi_{g}=\sin^{-1}(\frac{\lambda }{s})$$

This equation involves λ, the wavelength of the operating signal, and s, the spacing between neighboring CMUT cells. By maintaining s < λ/2, the grating lobes can be suppressed, ensuring reduced interference and improved imaging or signal reception.

The main lobe, which directs the strongest part of the beam, is characterized by its angle $\varphi_{x}$, calculated as follows [21]:

(7) $$\varphi_{x}=\sin^{-1}(\frac{\lambda }{L})$$

The angular position depends on the array’s total length (L) and the signal’s wavelength (λ). Extending the array length results in a narrower main lobe, thereby enhancing the resolution and reducing unwanted interference.

The main lobe angle is directly influenced by both the wavelength and the array length. At the lower frequency, with a longer wavelength, the main lobe is wider. In contrast, at the higher frequency, the shorter wavelength results in a much narrower main lobe, which provides higher directivity. The grating lobe angles depend on the cell spacing, s. By designing s < λ/2, unwanted lobes are suppressed, ensuring reduced interference and clearer imaging.

Optimizing the parameters ensures a sharp and well-focused beam, crucial for high-quality imaging or signal processing.

### 2.2. Fabrication of CMUT Array

**Figure 2:**
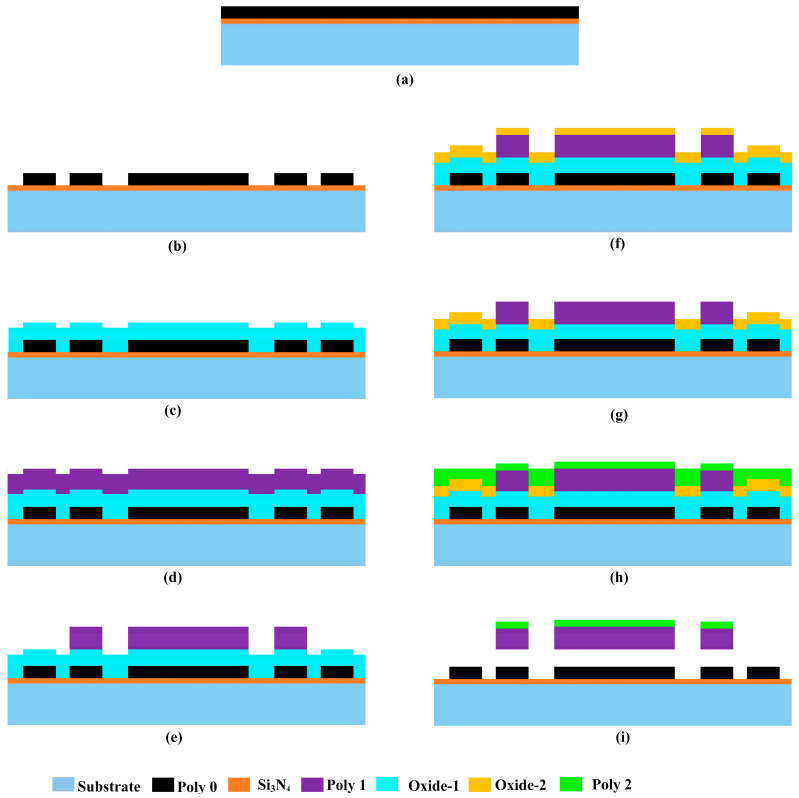
Cross-sectional illustrations showing the sequential fabrication steps of the MEMS CMUT developed through the PolyMUMPs microfabrication method. (**a**) Deposition of Si_3_N_4_ (600 nm) and Poly0 (500 nm) blanket. (**b**) Poly0 lithographically etched to form bottom electrodes. (**c**) Oxide-1 PSG (2 µm) deposited and anchor windows opened. (**d**) Poly1 structural layer (2 µm) and PSG mask deposited. (**e**) Poly1 patterned via PSG mask, defining membrane and posts. (**f**) Oxide-2 PSG sacrificial layer blanket-deposited and annealed. (**g**) Oxide-2 etched to create Poly1-to-Poly2 via holes. (**h**) Poly2 (1.5 µm) deposited, patterned, and fused to Poly1. (**i**) Sacrificial oxides HF-etched, releasing CMUT cavity and membrane.

The CMUT array was developed using the PolyMUMPs fabrication process, as outlined in [22]. This technique, depicted in Figure 2 for a single CMUT, incorporates polysilicon layers as the main structural components, while oxide layers serve as sacrificial materials to form cavities and create separation between the upper plate and the lower electrode. The process starts with 100 mm n-type (100) silicon wafers. Initially, a 600 nm layer of silicon nitride is deposited onto the substrate via low-pressure chemical vapor deposition (LPCVD). This layer functions as an effective electrical insulator and acts as a barrier to prevent impurities from penetrating the substrate.

Following this, a 500 nm polysilicon layer, referred to as Poly0, is deposited on top of the nitride layer (Figure 2a). Using photolithography and reactive ion etching (RIE), the Poly0 layer is patterned to establish the fixed bottom electrodes of the MEMS varactor (Figure 2b). A subsequent step involves depositing a 2 µm phosphosilicate glass (PSG) sacrificial layer, known as Oxide-1, through LPCVD. This layer is then annealed at 1050 °C for an hour in an argon environment. Oxide-1 is patterned and etched using RIE to define anchor structures (Figure 2c). The first structural polysilicon layer, Poly1, with a thickness of 2.0 µm, is then deposited over the oxide (Figure 2d), followed by a thin 200 nm PSG layer serving as a hard mask. The wafer is subjected to annealing at 1050 °C for an hour, allowing phosphorus diffusion from the PSG layers into the polysilicon, which helps in doping and stress reduction in Poly1. A lithographic mask is used to define Poly1 (Figure 2e), and the PSG layer is subsequently etched to act as a hard mask for further polysilicon etching. After this, the photoresist is stripped, and the oxide hard mask is removed via RIE.

A second sacrificial oxide layer is deposited and annealed on the wafer (Figure 2f). It is then patterned using the Poly1_Poly2_Via mask to create etch holes extending to the Poly1 layer, enabling mechanical and electrical connections between the Poly1 and Poly2 layers. RIE is then used to etch the oxide (Figure 2g). The second structural polysilicon layer, Poly2, with a thickness of 1.5 µm, is deposited (Figure 2h), followed by a 200 nm PSG layer. As in the previous step, the PSG layer functions as both a doping source and an etch mask. The wafer is then annealed again at 1050 °C for an hour, facilitating phosphorus diffusion into the polysilicon while mitigating residual stress in the Poly2 layer. The Poly2 layer is defined using a lithographic mask, followed by etching of both PSG and polysilicon under similar conditions as the Poly1 layer. After this step, the photoresist is stripped, and the oxide mask is removed. In areas where the Poly1_Poly2_Via pattern is defined, the Poly1 and Poly2 layers fuse, forming the final membrane structure.

To complete the fabrication, the wafer undergoes a final lithographic patterning step, and a 0.5 µm metal layer is deposited and structured using the liftoff method, which is specifically used for contact pads and interconnects. The last stage of the process involves releasing the structure (Figure 2i) by dissolving the sacrificial oxide layers with hydrofluoric acid (HF). The fabricated chips are then rinsed with deionized (DI) water, followed by alcohol, and subjected to critical point drying to eliminate stiction.

## 3. Results

### 3.1. COMSOL Simulation

**Figure 3:**
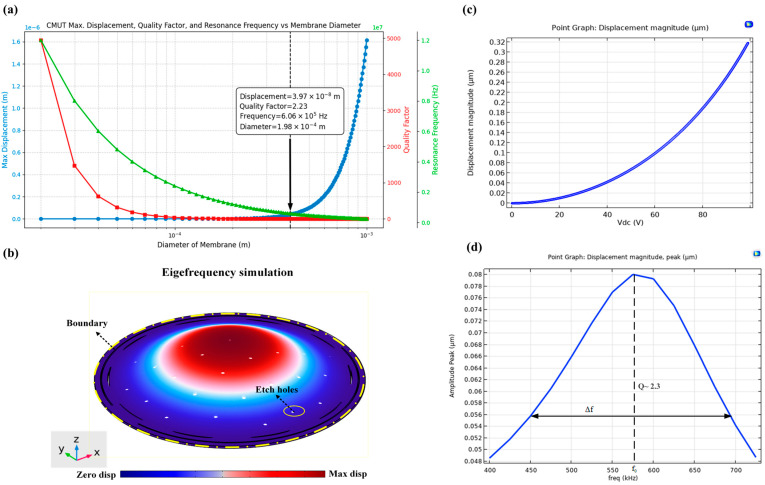
(**a**) Optimization plot of simulated metrics, (**b**) mode shape analysis, (**c**) displacement simulation based on different DC voltages applied, and (**d**) displacement frequency response simulation.

The structure was designed with a higher amplitude, lower quality factor, and higher frequency by incorporating the combined equations from Section 2, as shown in Figure 3a. COMSOL simulations were used to refine the design, focusing on optimizing the mechanical and acoustic performance of the CMUT to meet the specified parameters.

Thin-film damping simulations were conducted to model the squeeze-film effect between the vibrating membrane and the substrate (Figure 3b,c). These simulations provided the damping coefficient, membrane displacement, and pressure distribution, allowing for parameter adjustments, such as modifying the Q-factor, to achieve the intended CMUT performance. The applied voltage was set at 50 V, and the permittivity of free space was taken as 8.854 × 10^−12^ F/m.

Key design parameters included an initial capacitive gap height of 2 µm, polysilicon as the membrane material with a Young’s modulus of 160 GPa and a Poisson’s ratio of 0.22, and the incorporation of 33 etch holes in the diaphragm to comply with microfabrication design constraints. The results in Figure 3d indicate a low Q-factor of approximately 2 that is dominated by the squeeze-film damping effect observed in the design.

### 3.2. Fabricated Device

Based on the same optimization method, three more arrays were designed and fabricated along with the one described above.

**Figure 4:**
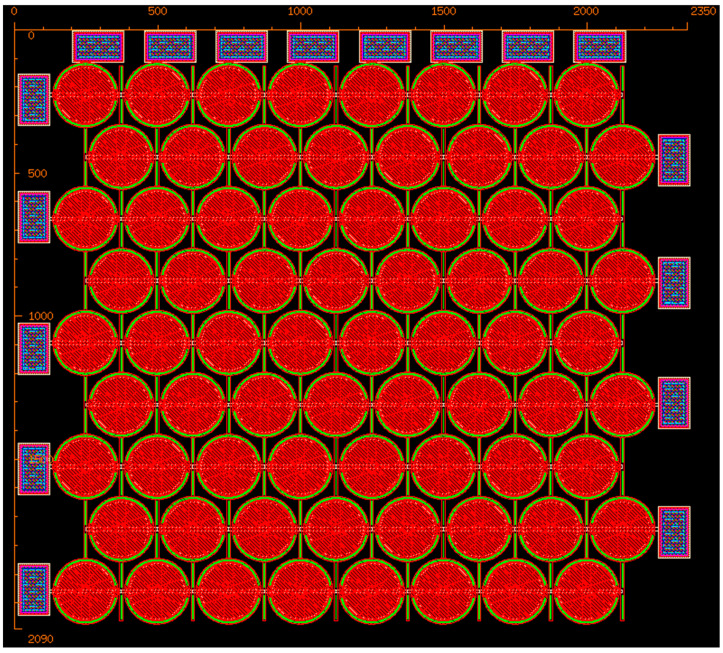
Layout of the proposed MEMS CMUT array design. Red-hatched discs are the Poly1 CMUT membranes sitting above black-white-hatched Poly0 cavity floors with brown dimples, magenta Poly2 overlies them, linked by cyan vias and green anchors, while red/black/magenta hole layers open etch paths for release. Peripheral blue-hatched metal stacks (with Poly2) form the bond pads, yellow guides mark rulers/keep-outs, and remaining palette colors are auxiliary anchors or unused mechanical layers.

The overall layout of the MEMS CMUT array in micrometer scale is illustrated in Figure 4. The Poly0 layer functions as a routing layer, facilitating the electrical connections between the capacitor plates and the contact pads. A 0.5 µm metal layer was deposited and precisely structured using the liftoff process to ensure proper metal interconnections. Further details on cell dimensions and spacing will be discussed in the next section.

**Figure 5:**
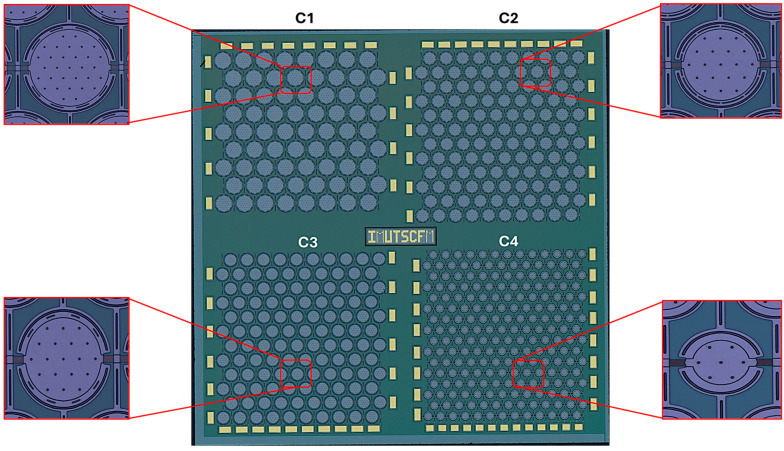
Fabricated device showing four CMUT array designs with insets showing zoomed-in views of the CMUT elements.

Figure 5 shows the microscope image captured by a Keyence VHX-7000N (KEYENCE Corporation, 1-3-14 Higashi-Nakajima, Higashi-Yodogawa-ku, Osaka 533-8555, Japan) from the four fabricated CMUT arrays. The diaphragm has a gap of 2 µm, and the holes are uniformly distributed with radius of r_0_ = 4 µm. The parameters for the CMUTs utilized in each array are provided in Table 1.

### 3.3. Imaging Simulation

**Figure 6:**
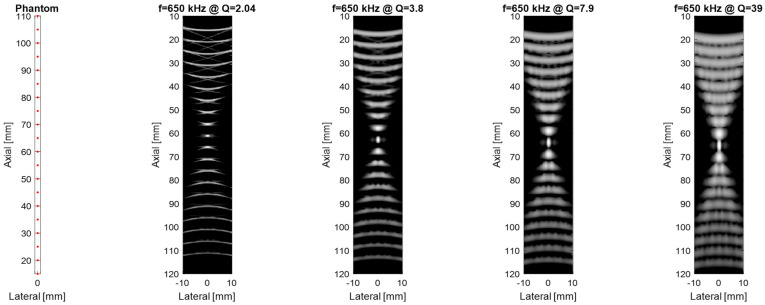
Comparison of imaging performance as a function of quality factor at a frequency (f_0_) of 650 kHz.

For the next part, the Field II ultrasound simulation program in Matlab R2021b [23] can be used to model the B-mode imaging process for different MEMS arrays by defining each transducer’s geometry, center frequency, bandwidth, and excitation pulse, and then placing scatterers in a phantom to simulate pulse-echo responses; to further analyze imaging performance, the point-spread function (PSF) is calculated by positioning point targets at regular intervals (e.g., every 5 mm starting at 15 mm from the transducer surface) and sweeping the beam over these points. This approach reveals how dynamic focusing, apodization schemes (e.g., Hanning windows), and transducer parameters, such as a 128-element aperture, 5 mm element height, element width equal to 1 wavelength, 0.1 mm kerf, and a 2-cycle Hanning-weighted signal, shape the PSF across different depths. When a linear sweep is performed on the point phantom and the resulting data is compressed to a 60 dB dynamic range, the spatial variation of the PSF becomes evident, illustrating trade-offs in resolution and penetration for transducers at different frequencies and Q-factors (where higher frequency improves resolution but increases attenuation, and lower Q-factor broadens bandwidth for sharper axial resolution but can reduce the surface image quality). In the context of Field II ultrasound simulations, a phantom is simply a collection of point scatterers placed at known coordinates. Each scatterer acts like a tiny reflector, with an assigned amplitude that determines how strongly it reflects incoming ultrasound. The number of scatterers (or “cells”) varies depending on how the phantom is defined. Some phantoms have only a few scatterers for simple tests, while others have thousands to simulate realistic tissue. Typically, the transducer (or “sensor”) lies in the y = 0 plane, facing along the positive y-axis so that the “axial” direction is y, and the “lateral” direction is x; therefore, scatterers in the phantom occupy positive y-values in front of the transducer face, allowing the simulation to compute echoes and form an ultrasound image in the lateral vs. axial coordinate space. The results from this Field II simulation are shown in Figure 6 and illustrate that decreasing the quality factor can enhance the axial resolution.

Figure 6 visualizes how the axial point-spread function (PSF) degrades when Q is allowed to rise while the center frequency is held at 650 kHz. Reducing Q from 39 to 2 collapses the ring-down time from 23 µs to 1.2 µs, shrinks the −6 dB axial width from 2.1 mm to 0.46 mm, and depresses the first side-lobe by 16 dB. This quantitative link between Q, τ and image quality underpins our target specification of Q ~ 2 for near-field airborne applications.

Another phantom is a 2D region of random scatterers (x = ±5 mm, y = 20–60 mm) with a 3 mm anechoic cyst at (0.40 mm). A 64-element linear transducer at z = 0 electronically scans across x = ±5 mm, transmitting and receiving ultrasound pulses. The resulting signals for each scan line are envelope-detected and log-compressed to form the B-mode images.

**Figure 7:**
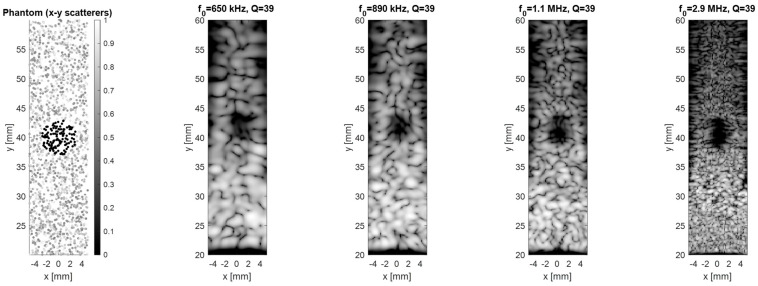
Comparison of imaging performance as a function of center frequency (f_0_) at a fixed high Q of 39.

Figure 7 shows results for sweeping the frequency while keeping Q fixed at ≈ 39. As expected from diffraction theory, the lateral full-width-half-maximum improves four-fold (2.6 to 0.55 mm) when frequency is raised from 650 kHz to 2.9 MHz, but the echo magnitude drops by ~8 dB due to absorption in air. Therefore, increasing the frequency enhances near-surface resolution, confirming that higher frequencies excel at shallow imaging while lower frequencies penetrate better at greater depths. The dual requirement for both long-range SNR and fine spatial detail therefore motivates the multi-frequency architecture implemented in this work.

### 3.4. Mode Shape

Actuation responsivity was measured as 0.77 ± 0.05 µm V^−1^ by scanning the membrane center with a Polytec OFV-552 laser vibrometer, Polytec GmbH, Waldbronn, Germany (10× objective), integrating the velocity output from the Polytec OFV-5000 decoder, and fitting displacement vs. applied voltage (30 V DC + 1 Vpp from a Tektronix AFG3122, Tektronix, Inc., Beaverton, OR, USA to Mini-Circuits ZFBT-4R2G-FT, Mini-Circuits (corporate HQ), Brooklyn, NY, USA bias-T to CMUT). Transmit sensitivity was measured as 130 ± 4 mPa V^−1^ (¼″ G.R.A.S. 46BE hydrophone, GRAS Sound & Vibration A/S, Holte, Denmark at 10 mm). 

The output signals were monitored using a PC (via VibSoft v4.8), while the LDV captured membrane displacement and vibration profiles in real time. An amplifier increased the signal power to ensure sufficient excitation levels.

**Figure 8:**
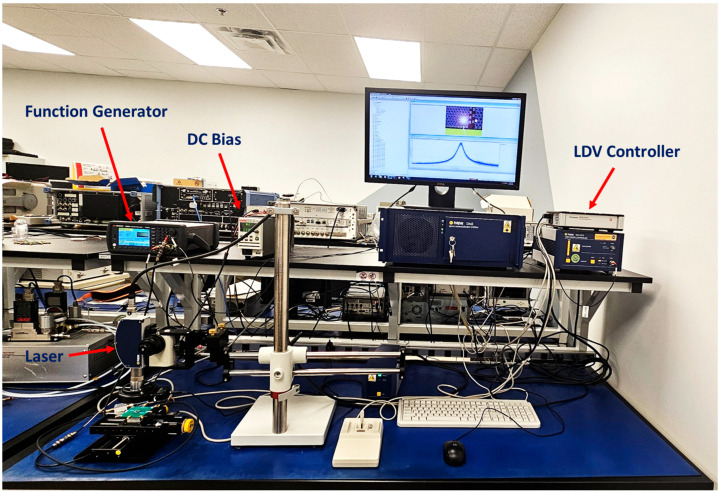
LDV setup with the device under test (DUT).

**Figure 9:**
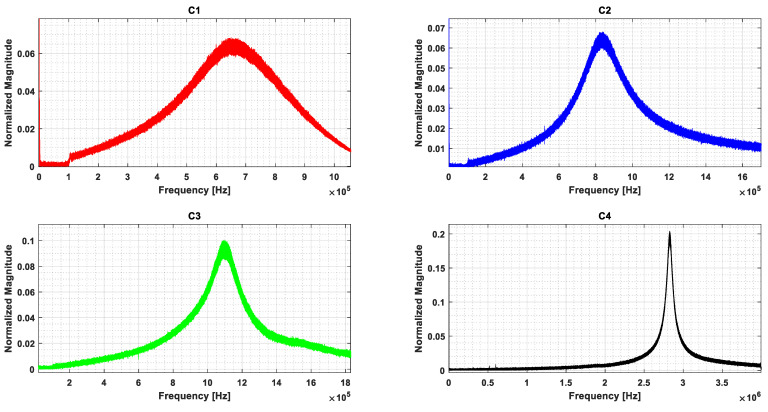
Experimental frequency responses for the array of CMUTs (C1, C2, C3 and C4) extracted using an LDV.

As shown in Figure 8, this setup enabled the characterization of resonance, displacement, and frequency response, facilitating the validation of the CMUT’s low-Q behavior and imaging performance. The measured vibration signals for four arrays are presented in Figure 9.

The graphical air-coupled mode shape results for the four arrays were measured by applying a DC voltage of 30 V and carrying out a scan with the LDV, as shown in Table 2.

This verifies the predicted displacement profiles and structural integrity of the membranes under oscillation.

### 3.5. Air-Coupled Ring-Down Q-Factor Measurement

Measurement results of ring-down airborne oscillations for the CMUTs were obtained to evaluate the Q-factor. The ring-down metrology is performed by driving the CMUT with a 30 V DC bias combined with a 1 Vpp rectangular pulse (Tektronix AFG3122 to Mini-Circuits ZFBT-4R2G-FT bias-T to CMUT under test), while out-of-plane velocity is captured by a Polytec OFV-552 laser head, decoded via OFV-5000, and digitized at 2 MS/s, 24 bit on a NI-PCI-5922 card, National Instruments, 11500 North Mopac Expressway, Austin, TX 78759, USA. The average 200 free-decay traces are recorded, the analytic envelope via Hilbert transform and fit log(envelope) versus time are extracted to obtain the 1/e decay constant τ. The oscillation measurements track signal decay and resonance behavior, confirming low-Q operation. Faster decay times indicate reduced ringing and improved temporal resolution, as described by (8):

Q = πfτ(8)

**Figure 10:**
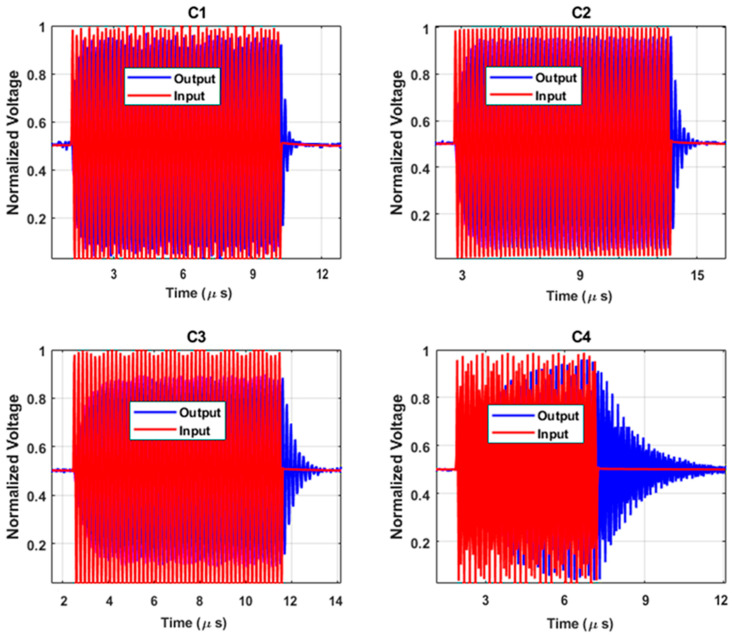
Air-coupled ring-down time measurement to evaluate the Q for each array.

In this equation, *f* represents the resonance frequency, and *τ* is the ring-down time constant related to damping effects such as squeeze-film damping [24]. The results in Figure 10 are based on a input signal with a period of 1 ms and an amplitude of 1 Vpp. Table 3 presents the results derived from the measured *τ* along with the simulated Q-factor.

In our CMUT design, the thin gap (e.g., 2 µm) between the vibrating membrane and the substrate causes the air to be rapidly compressed and decompressed. Because the device operates in air (with c ≈ 343 m/s) the squeeze-film damping becomes very effective at dissipating energy. The presence of perforations further enhances this effect by allowing air to escape more easily.

Factors such as the membrane material properties, gap height, hole numbers, perforation size and density, and operating frequency all interplay to reduce the Q-factor. Our modeling shows that a higher damping coefficient, which is directly related to these parameters, leads to a lower Q-factor.

A low Q-factor is advantageous in ultrasonic imaging because it minimizes prolonged oscillations (ringing). This results in sharper pulse responses and improved temporal resolution, which are critical for clear image formation and reducing side-lobe artifacts.

Our analysis confirms that the design’s emphasis on leveraging the squeeze-film damping effect, amplified by the optimized perforation pattern, is the primary reason behind achieving a low Q-factor. This ensures that the device produces clean, rapid transient responses ideal for high-resolution imaging.

### 3.6. Maximal Output

Devices were measured in ambient conditions of around 25 °C, 50%RH. The low-Q operation is robust for indoor and mild-outdoor conditions. For humid applications (i.e., 85%RH), the array may require a parylene-C conformal coating to block moisture ingress.

**Figure 11:**
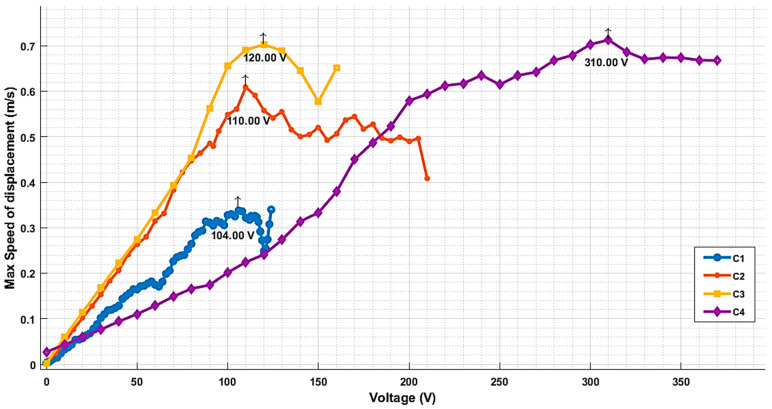
Output levels vs. drive voltage for the four CMUT cells.

The output signal strength under varying input DC voltages was measured until the collapse voltage was reached for each array, and the results are shown in Figure 11. Higher outputs correlated with optimized membrane geometries and damping characteristics. The results demonstrate the trade-off between displacement and the Q-factor, where higher-Q designs provided a higher maximal output.

While a higher output may be desirable for achieving a strong signal, it comes with the drawback of increased ringing, longer decay times, which can degrade temporal resolution. In contrast, our design aims to achieve a balance by reducing the Q-factor to minimize ringing, even if that means a lower maximal output.

Ultra-low-Q operation offers several compelling advantages for airborne and high-speed imaging applications. First, the reduced ring-down time (τ ≈ 1 µs) minimizes reverberations and enables pulse repetition frequencies up to 250 kHz, which are essential for high-frame-rate ultrasound imaging and airborne non-destructive testing (NDT). Second, the broader bandwidth of 75–80% significantly improves axial resolution by approximately threefold and allows for multi-frequency and harmonic imaging techniques. Third, side-lobe suppression is enhanced, as demonstrated by Field II simulations showing a 6 dB reduction in the first side-lobe energy compared to a higher-Q (Q ≈ 40) design. However, these advantages come with a trade-off: a reduction in acoustic displacement of approximately 30% at constant bias, which can lower transmit pressure (see Figure 11). To mitigate this, we implemented two effective strategies: (i) a four-stage low-noise preamplifier (2.3 nV/√Hz) was used to preserve SNR, and (ii) a transmit-only bias voltage boost to 70 V with a 1% duty cycle, remaining well below the measured collapse voltage. These measures allow the system to retain the benefits of ultra-low-Q performance and minimize the impact of the lower displacement.

### 3.7. Result Comparison

The proposed PolyMUMPs CMUT achieves an ultra-low fabrication cost (<$22/cm^2^) and CMOS-ready biasing (<100 V) with a low 100 V collapse voltage, tunable air Q of 2–40, an exceptionally wide 75–92 % fractional bandwidth, rapid 1.2 µs ring-down, and 4 on-die frequency bands, as shown in Table 4.

## 4. Conclusions

This study presented the design, fabrication, and performance evaluation of CMUT arrays optimized for low-Q operation, with one array achieving a Q-factor near 2. The developed device mitigated common issues associated with high Q-factors, such as prolonged ringing and side-lobe interference, leading to sharper main lobes and improved imaging resolution. The theoretical framework incorporated squeeze-film damping principles to tailor the mechanical and acoustic properties. FEA simulations confirmed the feasibility of the design, validating improvements in displacement, mode shape, and damping characteristics.

Experimental validation, including LDV measurements and ring-down oscillation tests, demonstrated strong agreement with simulation results. Fabricated using the PolyMUMPs process, the four array variants exhibited reliable operation and reduced signal decay time, making them suitable for air-coupled applications requiring high spatial and temporal resolution. Each array provided different signal output and Q-factor trade-offs. These findings highlight the potential of optimized designs for advancing ultrasonic imaging technologies and inform future research on array configurations and integration with imaging systems.

The implementation of multi-frequency CMUT arrays such as on the die proposed here can enhance imaging versatility across different airborne applications. By integrating multiple frequency bands onto one substrate such as that proposed here, adaptable imaging focus could be achieved, improving the suitability of CMUT arrays for diverse diagnostic scenarios.

Future work could explore adaptive focusing and control using machine learning to dynamically optimize parameters across arrays for diverse air-coupled applications. Real-time integration with AI-driven processing may further enhance noise suppression and diagnostic accuracy. Additionally, the development of energy-efficient devices with self-tuning capabilities could optimize power consumption while maintaining high performance, making them suitable for portable applications.

## Figures and Tables

**Table 1:** CMUT parameters for the four array variants.

	Diameter (µm)	Frequency (MHz)	Anchor Width (µm)	Holes Numbers	Q	Velocity (mm/s)
C1	200	0.65	20	33	2.04	177.38
C2	150	0.84	30	19	3.98	339.62
C3	150	1.12	2.5	19	7.66	552.30
C4	85	2.91	30	7	39.41	939.42

**Table 2:** Airborne mode shape image by LDV for the four different CMUT arrays.

Array Angle	C1	C2	C3	C4
**0**	* 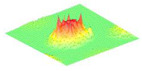 *	* 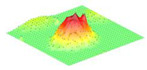 *	* 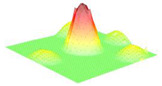 *	* 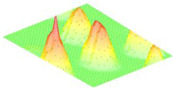 *
**45**	* 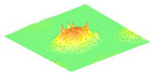 *	* 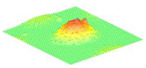 *	* 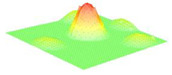 *	* 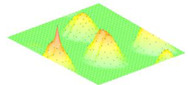 *
**90**	* 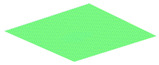 *	* 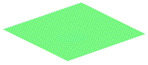 *	* 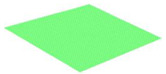 *	* 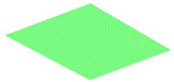 *
**135**	* 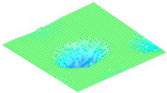 *	* 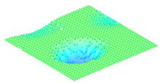 *	* 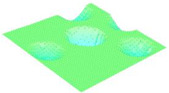 *	* 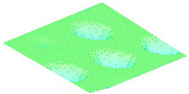 *
**180**	* 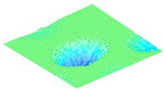 *	* 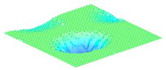 *	* 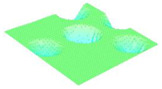 *	* 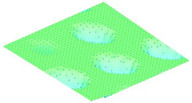 *
**225**	* 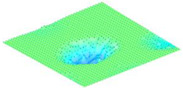 *	* 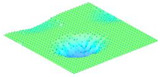 *	* 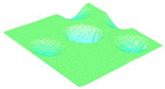 *	* 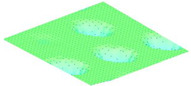 *
**270**	* 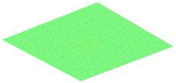 *	* 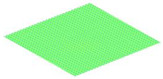 *	* 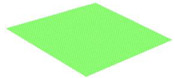 *	* 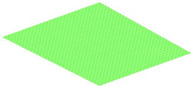 *
**315**	* 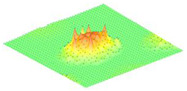 *	* 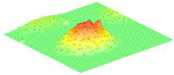 *	* 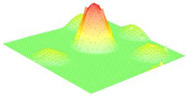 *	* 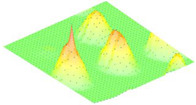 *

**Table 3:** Ring oscillation results for four arrays.

	Measured τ (µs)	Measured Q from Ring-down Test	Simulated Q from FEA
C1	1.19	2.44	2.04
C2	1.58	4.17	3.98
C3	2.06	7.23	7.66
C4	4.57	41.75	39.41

**Table 4:** Result comparison for the CMUT designs.

Metric	This Work	Bayram 2005 [25]	Demirci 2004 [3]	Goel 2022 [12]	Annayev 2025 [26]	Wang 2024 [6]
Fabrication and cost	3-mask PolyMUMPs; <$22 cm^−2^	SOI bonding; ≈$120 cm^−2^	SOI bonding; ≈$130 cm^−2^	PolyMUMPs; ≈$25 cm^−2^	3-mask glass-bond; ≈$90 cm^−2^	SOI; ≈$145 cm^−2^
Collapse voltage (V)	100 V	177 V	155 V	110 V	35 V (pre-charged)	210 V
Max.displacement (µm)	0.33 ± 0.02 @ 80 V (LDV)	0.95 µm	Not reported	0.025 µm	Not reported	Not reported
Q-factor (air)	2–40 (tunable)	30–80	22–45	35 ± 7	25 ± 4	25
−6 dB fractional BW	75–92%	20–25%	35%	25%	34%	30%
Ring-down τ (µs)	1.2	12–18	6–9	7–10	8.5	8
Multi-band on-die	4 bands	No	No	No	No	No
CMOS-ready bias (≤100 V)	Yes	No	No	Yes	Yes	No

## Data Availability

The original contributions presented in the study are included in the article, further inquiries can be directed to the corresponding author.
